# Milk kefir: composition, microbial cultures, biological activities, and related products

**DOI:** 10.3389/fmicb.2015.01177

**Published:** 2015-10-30

**Authors:** Maria R. Prado, Lina Marcela Blandón, Luciana P. S. Vandenberghe, Cristine Rodrigues, Guillermo R. Castro, Vanete Thomaz-Soccol, Carlos R. Soccol

**Affiliations:** ^1^Department of Bioprocess Engineering and Biotechnology, Federal University of ParanáCuritiba, Brazil; ^2^Nanobiomaterials Laboratory, Institute of Applied Biotechnology – School of Sciences, Universidad Nacional de la PlataLa Plata, Argentina

**Keywords:** kefir, biological activity, polysaccharides, kefiran, microbial composition

## Abstract

In recent years, there has been a strong focus on beneficial foods with probiotic microorganisms and functional organic substances. In this context, there is an increasing interest in the commercial use of kefir, since it can be marketed as a natural beverage that has health promoting bacteria. There are numerous commercially available kefir based-products. Kefir may act as a matrix in the effective delivery of probiotic microorganisms in different types of products. Also, the presence of kefir’s exopolysaccharides, known as kefiran, which has biological activity, certainly adds value to products. Kefiran can also be used separately in other food products and as a coating film for various food and pharmaceutical products. This article aims to update the information about kefir and its microbiological composition, biological activity of the kefir’s microflora and the importance of kefiran as a beneficial health substance.

## Introduction

Kefir is an acidic-alcoholic fermented milk product with little acidic taste and creamy consistency that was originated in the Balkans, in Eastern Europe, and in the Caucasus (Fontán et al., 2006; Serafini et al., 2014). Kefir can be produced by fermenting milk with commercial freeze-dried kefir starter cultures, traditional kefir grains, and the product that remains after the removal of kefir grains (Bensmira et al., 2010). Kefir grains are a kind of yogurt starter, which are white to yellow – white, gelatinous, and variable in size (varying from 0.3–3.5 cm in diameter) and are composed by a microbial symbiotic mixture of lactic acid bacteria (10^8^ CFU/g), yeast (10^6^–10^7^ CFU/g), and acetic acid bacteria (10^5^CFU/g) that stick to a polysaccharide matrix (Garrote et al., 2010; Chen et al., 2015). After successive fermentations, kefir grains can break up to new generation grains, which have the same characteristics as the old ones (Gao et al., 2012).

Commercial kefir is produced by two methods: The “Russian method” and the pure cultures. In the “Russian method” kefir is produced on a larger scale, using a series fermentation process, beginning with the fermentation of the grains and using the percolate. The other method employs pure cultures isolated from kefir grains or commercial cultures (Leite et al., 2013). Also, the industrial or commercial process uses direct-to-vat inoculation (DVI) or direct-to-vat set (DVS) kefir starter cultures. In addition, *Bifidobacterium* sp., *Lactobacillus* sp. and probiotic yeast (*Saccharomyces boulardii*) may be used as adjunct cultures when blended with kefir grains or kefir DVI cultures (Wszolek et al., 2006). On the other hand, whey may be a practical base for kefir culture production, and fermented whey has shown to be a suitable cryoprotective medium during freeze-drying. The freeze-dried culture retains a high survival rate and shows good metabolic activity and fermentation efficiency, indicating a good potential for its use as a value-added starter culture in dairy technology. All of these studies have shown promising perspectives for the application of kefir grains in whey valorization strategies (Bensmira et al., 2010; Cheirsilp and Radchabut, 2011).

Traditionally, kefir is manufactured using cow, ewe, goat, or buffalo milk. However, in some countries, animal milk is scarce, expensive, or minimally consumed due to dietary constraints, preferences, or religious customs. Therefore, there have been many attempts to produce kefir from a variety of food sources such as soy milk (Botelho et al., 2014). Historically, kefir has been linked with health, for example, in Soviet countries, kefir has been recommended for consumption by healthy people to restrain the risk of some diseases (Saloff-Coste, 1996; St-Onge et al., 2002; Farnworth and Mainville, 2003). The consumption of this fermented milk has been related to a variety of health benefits (Vujičič et al., 1992; McCue and Shetty, 2005; Rodrigues et al., 2005a) not only linked to its microflora, but also due to the presence of some metabolic products as organic acids (Garrote et al., 2001; Ismaiel et al., 2011). In addition, kefir cultures have the ability to assimilate cholesterol in milk (Yanping et al., 2009). On the other hand, there is a growing commercial interest in using kefir as a suitable food matrix for supplementation with health-promoting bacteria. Kefir may not only be a natural probiotic beverage, but also acts as an effective matrix for the delivery of probiotic microorganisms (Vinderola et al., 2006; Medrano et al., 2008; Oliveira et al., 2013).

In kefir grains the main polysaccharide is kefiran, which is a heteropolysaccharide composed by equal proportions of glucose and galactose and is mainly produced by *Lactobacillus kefiranofaciens* (Zajšek et al., 2011). It has been demonstrated that kefiran improves the viscosity and viscoelastic properties of acid milk gels (Rimada and Abraham, 2006), and is able to form gels that have interesting viscoelastic properties at low temperatures, because of that, kefiran can also be used as an additive in fermented products. Besides, kefiran can enhance the rheological properties of chemically acidified skim milk gels increasing their apparent viscosity (Zajšek et al., 2013).

Compared with other polysaccharides, kefiran has outstanding advantages such as antitumor, antifungal, antibacterial properties (Cevikbas et al., 1994; Wang et al., 2008) immunomodulation or epithelium protection (Serafini et al., 2014), anti-inflammatory (Rodrigues et al., 2005b), healing (Rodrigues et al., 2005a), and antioxidant activity (Chen et al., 2015).

This review presents the most recent advances about kefir and kefiran, their production and microbial cultures involved, biological activities and potential applications in health and food industries.

## Microbial Composition of Kefir Grains and Kefir

Kefir grains have a complex composition of microbial species such as the predominance of lactic acid bacteria, acetic bacteria, yeasts, and fungi (Jianzhong et al., 2009; Pogačić et al., 2013). This microbial species are classified into four groups: homofermentative and heterofermentative lactic acid bacteria and lactose and non-lactose assimilating yeast (Cheirsilp and Radchabut, 2011). In that way, *Lactobacillus paracasei* ssp. *paracasei*, *Lactobacillus acidophilus, Lactobacillus delbrueckii* ssp. *bulgaricus*, *Lactobacillus plantarum*, and *L. kefiranofaciens* are predominant species. However, these species represent only 20% of the *Lactobacillus* in the final fermented beverage, with the remainder consisting of *Lactobacillus kefiri* (80%; Yüksekdag et al., 2004; Zanirati et al., 2015). *Acetobacter aceti* and *A. rasens* have also been isolated, such as the fungus *Geotrichum candidum*. More than 23 different yeast species have been isolated from kefir grains and from fermented beverages of different origins. However, the predominant species are *Saccharomyces cerevisiae, S. unisporus, Candida kefyr*, and *Kluyveromyces marxianus* ssp. *marxianus* (Witthuhn et al., 2004; Diosma et al., 2014; Zanirati et al., 2015; **Table 1**).

**Table 1:** Microbial compositions found in kefir and kefir grains of different origins.

Microorganism	Source – Country	Reference
*Lactobacillus kefir, Lactobacillus kefiranofaciens, Lactobacillus paracasei, Lactobacillus plantarum, Lactococcus lactis* ssp. *lactis, Kluyveromyces marxianus, Lactobacillus parakefir, Saccharomyces cerevisiae, Saccharomyces unisporus, Leuconostoc mesenteroides, Acetobacter* sp., *Saccharomyces* sp., *Lactococcus lactis* ssp. *lactis* biovar *diacetylactis*, *Lactococcus lactis, Lactobacillus kefiri, Lactobacillus parakefiri*	Kefir grains and beverage – Argentina	Garrote et al., 2001; Londero et al., 2012; Hamet et al., 2013; Diosma et al., 2014.
*Lactobacillus kefiri, Lactobacillus kefiranofaciens, Leuconostoc mesenteroides, Lactococcus lactis, Lactococcus lactis* ssp. *cremoris, Gluconobacter frateurii, Acetobacter orientalis, Acetobacter lovaniensis, Kluyveromyces marxianus, Naumovozyma* sp., *Kazachastania khefir*	Kefir grains and beverage – Belgium	Korsak et al., 2015
*Lactobacillus kefiri, Lactobacillus kefiranofaciens, Leuconostoc mesenteroides, Lactococcus lactis, Lactobacillus paracasei, Lactobacillus helveticus, Gluconobacter japonicus, Lactobacillus uvarum, Acetobacter syzygii, Lactobacillus satsumensis, Saccharomyces cerevisiae., Leuconostoc* sp., *Streptococcus* sp., *Acetobacter* sp., *Bifidobacterium* sp., *Halococcus* sp., *Lactobacillus amylovorus, Lactobacillus buchneri, Lactobacillus crispatus, Lactobacillus kefiranofaciens* ssp. *kefiranofaciens, Lactobacillus kefiranofaciens* ssp. *kefirgranum, Lactobacillus parakefiri*	Kefir grains – Brazil	Miguel et al., 2010; Leite et al., 2012; Zanirati et al., 2015
*Lactobacillus brevis, Lactobacillus delbrueckii* ssp. *bulgaricus, Lactobacillus helveticus, Streptococcus thermophilus, Lactobacillus casei* ssp. *pseudoplantarum, Kluyveromyces marxianus* var. *lactis, Saccharomyces cerevisiae, Candida inconspicua, Candida maris, Lactobacillus lactis* ssp. *lactis*	Kefir grains and beverage – Bulgaria	Simova et al., 2002
*Lactobacillus paracasei, Lactobacillus parabuchneri, Lactobacillus casei, Lactobacillus kefiri, Lactococcus lactis, Acetobacter lovaniensis, Kluyveromyces lactis, Kazachstania aerobia, Saccharomyces cerevisiae, Lachancea meyersii*	Kefir beverage – Brazil	Magalhães et al., 2011
*Lactobacillus kefiranofaciens, Leuconostoc mesenteroides, Lactococcus lactis, Lactobacillus helveticus, Kluyveromyces marxianus, Saccharomyces cerevisiae, Pseudomonas* sp.*, Kazachstania unispora, Kazachstania exigua, Lactobacillus kefiri, Lactobacillus casei, Bacillus subtilis, Pichia kudriavzevii, Leuconostoc lactis, Lactobacillus plantarum, Acetobacter fabarum, Pichia guilliermondii, Lactococcus* sp., *Lactobacillus* sp., *Acetobacter* sp., *Shewanella* sp., *Leuconostoc* sp., *Streptococcus* sp, *Acinetobacter* sp., *Pelomonas* sp., *Dysgonomonas* sp., *Weissella* sp., *Shewanella* sp.	Kefir grains (Tibet)– China	Jianzhong et al., 2009; Gao et al., 2012, 2013a
*Acetobacter acetic, Enterococcus faecalis, Enterococcus durans, Lactococcus lactis* ssp. *cremoris, Leuconostoc pseudomesenteroides, Leuconostoc paramesenteroides, Lactobacillus brevis, Lactobacillus acidophilus, Saccharomyces* sp., *Brettanomyces* sp., *Candida* sp., *Saccharomycodes* sp., *Acetobacter rancens*	Kefir beverage – China	Yang et al., 2007
*Lactobacillaceae* and *Streptococcaceae*	Kefir grains and beverage – Ireland	Dobson et al., 2011
*Lactobacillus kefiranofaciens, Dekkera anomala, Streptococcus thermophilus, Lactococcus lactis, Acetobacter* sp.*, Lactobacillus lactis, Enterococcus* sp., *Bacillus* sp., *Acetobacter fabarum, Acetobacter lovaniensis, Acetobacter orientalis*	Kefir grains – Italy	Garofalo et al., 2015
*Leuconostoc* sp.*, Lactococcus* sp.*, Lactobacillus* sp., *Lactobacillus plantarum, Zygosaccharomyces* sp.*, Candida* sp.*, Candida lambica, Candida krusei, Saccharomyces* sp., *Cryptococcus* sp.	Kefir grains and beverage – South Africa	Witthuhn et al., 2005
*Lactobacillus* sp., *Leuconostoc* sp., *Lactococcus* sp., *Zygosaccharomyces* sp., *Candida* sp., *Saccharomyces* sp.	Kefir grains – South Africa	Witthuhn et al., 2004
*Lactobacillus kefiri, Lactobacillus kefiranofaciens, Leuconostoc mesenteroides, Lactococcus lactis, Escherichia coli, Pseudomonas* sp., *Saccharomyces turicensis*,	Kefir grains – Taiwan	Wyder et al., 1999; Chen et al., 2008; Wang et al., 2012;
*Lactobacillus kefiri, Leuconostoc mesenteroides, Lactococcus lactis, Streptococcus thermophilus, Lactobacillus kefiranofaciens, Lactobacillus acidophilus*	Kefir grains and beverage – Turkey	Guzel-Seydim et al., 2005; Kesmen and Kacmaz, 2011
*Lactobacillus helveticus, Lactobacillus buchneri, Lactobacillus kefiranofaciens, Lactobacillus acidophilus, Lactobacillus helveticus, Streptococcus thermophilus, Bifidobacterium bifidum, Kluyveromyces marxianus*	Kefir grains – Turkey	Kok-Tas et al., 2012; Nalbantoglu et al., 2014
*Lactococcus cremoris, Lactococcus lactis, Streptococcus thermophilus, Streptococcus durans*	Kefir beverage – Turkey	Yüksekdag et al., 2004

The microbial composition may vary according to kefir origin, the substrate used in the fermentation process and the culture maintenance methods. Tibetan kefir, which is used in China, is composed of *Lactobacillus, Lactococcus*, and yeast. Additionally, acetic acid bacteria have been identified in Tibetan kefir, depending on the region in China from where it was obtained (Gao et al., 2012), additionally, Tibetan kefir composition differs from that of Russian kefir, Irish kefir, Taiwan kefir, Turkey fermented beverage with kefir; however, it is known that this microbial diversity is responsible for the physicochemical features and biological activities of each kefir (Jianzhong et al., 2009; Kabak and Dobson, 2011; Gao et al., 2012; Altay et al., 2013).

Wang et al. (2012) examined a section of a whole kefir grain and found in the outer layer of the grain, lactococci, and yeasts, and, in the inner layer of the grain, the quantity of lactobacilli were much higher and more yeasts cells were found. There are little information about the mechanism of grain formation, so the same authors, proposed a hypothesis to explain that. “Initially, *Lactobacillus kefiranofaciens* and *Saccharomyces turicensis* start to auto-aggregate and co-aggregated to small granules.” The aggregation is enhanced when the pH drops. The biofilm producers, *Lactobacillus kefiri*, *Kluyveromyces marxianus* HY1, and *Pichia fermentans* HY3 then adhere to the surface of these small granules due to their cell surface properties and their strong aggregation ability, which gives rise to thin biofilms. After biofilm formation, the kefir yeasts and *Lactobacillus* continue to co-aggregated with the granule strains and associate with the granule biofilm to become a three dimensional microcolony. As the cell density due to the growth of kefir yeasts and *Lactobacillus* increases, cells and milk components that are present in the liquid phase accumulate on the granule surface and the kefir grains are formed. There is a symbiotic relation between the microorganisms present in kefir grains, wherein the bacteria and yeast survive and share their bioproducts as power sources and microbial growth factors. This microorganism association is responsible for lactic and alcoholic fermentation (Witthuhn et al., 2005; Wang et al., 2012; Hamet et al., 2013).

After receiving its actual/present denomination, some of the microorganisms isolated and identified in kefir cultures were classified using the product name, as in *Lactobacillus kefiri, L. kefiranofaciens, L. kefirgranum, Lactobacillus parakefir*, and *Candida kefyr* (Wyder et al., 1999; Kwon et al., 2003; Yang et al., 2007; Kok-Tas et al., 2012). **Table 1** demonstrates the microbial composition, which has been isolated from kefir and kefir grains of different origins.

## Biological Activity of Kefir

Due to its composition, kefir is mainly considered a probiotic resource (Nalbantoglu et al., 2014). “Probiotics are microbial cell preparations or components of microbial cells with a beneficial effect on the health of the host” (Lopitz et al., 2006). Some studies suggest that probiotic bacteria in kefir consumers’ gut are abundant and are correlated with health improvement (Ahmed et al., 2013; Zheng et al., 2013); in that way, it had been demonstrated that the cell-free fraction of kefir enhances the ability to digest lactose relieving symptoms (Farnworth, 2005; Rizk et al., 2009).

Another reason for the increased interest in probiotic strains from kefir is its capacity to lower cholesterol levels. There are different ways in which bacteria can alter serum cholesterol: (i) through the binding to and absorption into the cell before it can be absorbed into the body; (ii) producing free and deconjugating bile acids; (iii) inhibiting the enzyme HMG-CoA reductase (Yanping et al., 2009).

The microorganisms in the kefir grains produce lactic acid, antibiotics and bactericides, which inhibit the development of degrading and pathogenic microorganisms in kefir milk (Liu et al., 2002). Kefir acts against the pathogenic bacteria *Salmonella, Helicobacter, Shigella, Staphylococcus, Escherichia coli, Enterobacter aerogenes, Proteus vulgaris, Bacillus subtilis, Micrococcus luteus, Listeria monocytogenes, Streptococcus pyrogenes*, (Lopitz et al., 2006), *Streptococcus faecalis* KR6, *Fusarium graminearum* CZ1 (Ismaiel et al., 2011), and the fungus *Candida albicans*. On the other hand, it has been demonstrated that a mixture of kefir isolated bacteria and yeast is able to prevent diarrhea and enterocolitis triggered by *Clostridium difficile* (Bolla et al., 2013). Besides, kefir showed good efficacy in inhibiting spore formation and aflatoxin B1 produced by the fungus *Aspergillus flavus*, which is a toxic compound formed either in the field or during food storage. Therefore, kefir appears as a promising safe alternative natural food preservative offering protection against intoxication with aflatoxin B1 (Ismaiel et al., 2011).

It had been proved that many species of lactobacilli present in kefir have S-layer proteins. Surface layers (S-layers) can be aligned in unit cells on the outermost surface of many prokaryotic microorganisms (Mobili et al., 2009). It has been demonstrated that these S-layer proteins can apply a protective action inhibiting the grown of *Salmonella enterica* serovar *Enteritidis* in Caco-2 cells, and also have the ability to antagonize the effects of toxins from *Clostridium difficile* on eukaryotic/eukaryotic cells *in vitro* (Carasi et al., 2012).

However, there are other important bioactivities that have been tested with kefir grains, the cell-free fraction of kefir or acid lactic bacteria isolated from kefir, such as antitumoral (Gao et al., 2013b), anti-inflammatory (Diniz et al., 2003), antimicrobial (Anselmo et al., 2010) immunoregulatory (Hong et al., 2009), antiallergenic (Wei-Sheng et al., 2010), wound healing (Huseini et al., 2012), antidiabetic (Young-In et al., 2006) antimutagenic (Guzel-Seydim et al., 2006), and antigenotoxic (Grishina et al., 2011). In that way, it had been demonstrated that kefir cell-free fraction has antiproliferative effects on human gastric cancer SGC7901 cells (Gao et al., 2013b), colon adenocarcinoma cells (Khoury et al., 2014), HuT–102 malignant T lymphocytes, sarcoma 180 in mice, Lewis lung carcinoma and human mammary cancer (Rizk et al., 2009), and reduce oxidative stress (Punaro et al., 2014). Another study has shown that suspensions after 24 h fermentation and mechanically disintegrated kefir grains cause a significant inhibition of granuloma tissue formation and a 43% inhibition of the inflammatory process (Diniz et al., 2003).

Nevertheless, there are other important studies performed with some microorganisms isolated from different types of kefir. Some microorganisms with their biological activities and origin are shown in **Table 2**.

**Table 2:** Kefir microorganisms and their biological activities.

Organism of interest	Origin	Biological activity	Reference
*Lactobacillus plantarum* MA2	Tibetan kefir	Hypocholesterolemic effect	Yanping et al., 2009
*Lactobacillus plantarum* Lp27	Tibetan kefir	Inhibited cholesterol absorption	Ying et al., 2013
*Lactobacillus plantarum* CIDCA 83114	Kefir grains – Argentina	Inhibit the growth of *Shigella sonnei in vitro* and also the cytotoxicity of *C. difficile* toxins on eukaryotic cells	Bolla et al., 2013
*Lactobacillus kefir* CIDCA 8348	Kefir grains – Argentina	Inhibit the growth of *Shigella sonnei in vitro* and also the cytotoxicity of *C. difficile* toxins on eukaryotic cells	Bolla et al., 2013
*Lactobacillus plantarum* ST8KF	Kefir grains – South Africa	Bactericida effect against: *Lactobacillus casei*, *Lactobacillus salivarius*, *Lactobacillus curvatus*, *Listeria innocua*	Powell et al., 2007
*Lactobacillus kefiranofaciens* K1	Kefir grains – Taiwanese milk	Antiallergenic effect	Chen et al., 2008; Wei-Sheng et al., 2010
*Lactobacillus kefiranofaciens* M1	Kefir grains – Taiwanese milk	Immunoregulatory effects – anticolitis effect	Hong et al., 2009; Chen et al., 2012
*Lactobacillus lactis* CIDCA 8221	Kefir grains – Argentina	Inhibit the growth of *Shigella sonnei in vitro* and also the cytotoxicity of *Clostridium difficile* toxins on eukaryotic cells	Bolla et al., 2013
*Kluyveromyces marxianus* CIDCA 8154	Kefir grains – Argentina	Inhibit the growth of *Shigella sonnei in vitro* and also the cytotoxicity of *Clostridium difficile* toxins on eukaryotic cells	Bolla et al., 2013
*Saccharomyces cerevisiae* CIDCA 8112	Kefir grains – Argentina	Inhibit the growth of *Shigella sonnei in vitro* and also the cytotoxicity of *Clostridium difficile* toxins on eukaryotic cells	Bolla et al., 2013
*Lactobacillus lactis* ssp. *cremoris*	Kefir grains – India	Activity against food spoilage bacteria	Raja et al., 2009

*Source: Soccol et al., 2014*.

## Kefiran, A Potential Exopolysaccharide

The increased search for natural polysaccharides has been very significant due to their use in the food, pharmaceutical, and cosmetic industries as additives, bio-absorbents, metal removal agents, bioflocculants, and medicine delivery agents, among other functions (De Vuyst et al., 2001; Welman and Maddox, 2003; Badel et al., 2011). Many microorganisms, such as bacteria, fungi, and weeds, have the capacity/ability to synthesize and excrete extracellular polysaccharides, and these polysaccharides can be either soluble or insoluble (Wang et al., 2010; Badel et al., 2011).

The polysaccharides that are commonly used as food additives are xanthan, dextran, gellan, and alginates, while the exopolysaccharides (EPSs) produced by lactic acid bacteria show good physicochemical characteristics for their use as food additives. In addition to these characteristics, EPSs are obtained from microorganisms classified as GRAS (generally recognized as safe), such as lactic acid bacteria (Wang et al., 2008; Saija et al., 2010; Badel et al., 2011).

Many reports have demonstrated that the quantity and properties of EPSs depend on the microorganisms used in the fermentation process and on the fermentation conditions and the composition of the culture media (Kim et al., 2008). EPSs have physicochemical and rheological properties that make them suitable as additives, which can be used as stabilizers, emulsifiers, gelling agents, and viscosity improvers. Additionally, EPSs possess biological properties suggesting their use as antioxidants, antitumor agents, antimicrobial agents, and immunomodulators, among other roles (Suresh Kumar et al., 2008; Bensmira et al., 2010; Piermaria et al., 2010).

The EPS kefiran is produced by *Lactobacillus kefiranofaciens* (Kooiman, 1968; Wang et al., 2010) from kefir grains, which are composed of proteins, polysaccharides, and a complex symbiotic microbial mixture (Witthuhn et al., 2005; Jianzhong et al., 2009). These microorganisms grow in kefiran, which is a polysaccharide matrix consisting of glucose and galactose. Despite good kefiran production by *L. kefiranofaciens* alone, it has been observed that the addition of *Saccharomyces* sp. to the culture improves the net quantity of kefiran, illustrating the importance of the symbiosis between the bacteria and yeast that are present in kefir (Cheirsilp et al., 2003).

Lactic acid bacteria can synthesize homopolysaccharides or heteropolysaccharides. The synthesized homopolysaccharides are glucans or fructans, which are composed of only one type of monosaccharide (glucose or fructose, respectively; Van Hijum et al., 2006; Badel et al., 2011), whereas the heteropolysaccharides contain different types of monosaccharides in different proportions (mainly glucose, galactose, and rhamnose), (De Vuyst and Degeest, 1999; Ruas-Madiedo et al., 2002).

Similarly to lactic acid bacteria, *Lactobacillus* sp. also produces glucan and fructan. The homopolysaccharides show a much higher performance compared with heteropolysaccharide production (Welman and Maddox, 2003; Badel et al., 2011).

The heteropolysaccharides excreted by *Lactobacillus delbrueckii*, *Lactobacillus bulgaricus*, *Lactobacillus rhamnosus*, and *Lactobacillus helveticus* contain galactose, glucose, and rhamnose as the main monosaccharides, with other monosaccharides being present in smaller concentrations. They are also highly branched with different types of linkages, and their denominations are complex and generally dependent on the main monosaccharide (De Vuyst and Degeest, 1999; Badel et al., 2011).

*Lactobacillus plantarum* isolated from Tibetan kefir excretes EPS classified as heteropolysaccharides composed of galactose, glucose, and mannose. This EPS has the capacity/ability to reduce blood cholesterol and form a biofilm shape (Zhang et al., 2009; Wang et al., 2010).

Kefiran is an EPS classified as a heteropolysaccharide comprising glucose and galactose in high concentrations, and it is classified as a water-soluble glucogalactan, which makes it suitable to be used as an additive (Wang et al., 2008, 2010). Kefiran has excellent rheological properties and can significantly improve the viscosity of lacteous products by favoring and maintaining gel properties and avoiding the loss of water during storage (Rimada and Abraham, 2006). With respect to the biological activity of kefiran, several studies have demonstrated that this EPS can be used as a nutraceutical, as described in **Table 3**.

**Table 3:** Biological activity of kefiran.

Exopolysaccharide	Biological activity	Reference
Kefiran	Reduction of blood pressure induced by hypertension	Maeda et al., 2004
	Favors the activity of peritoneal macrophages	
	Increase in peritoneal IgA	Duarte et al., 2006
	Antitumoral activity	Liu et al., 2002
	Antimicrobial activity	Rodrigues et al., 2005a
	Modulation of the intestinal immune system and protection of epithelial cells against *Bacillus cereus* exocellular factors	Medrano et al., 2008; Piermaria et al., 2010

**FIGURE 1:**
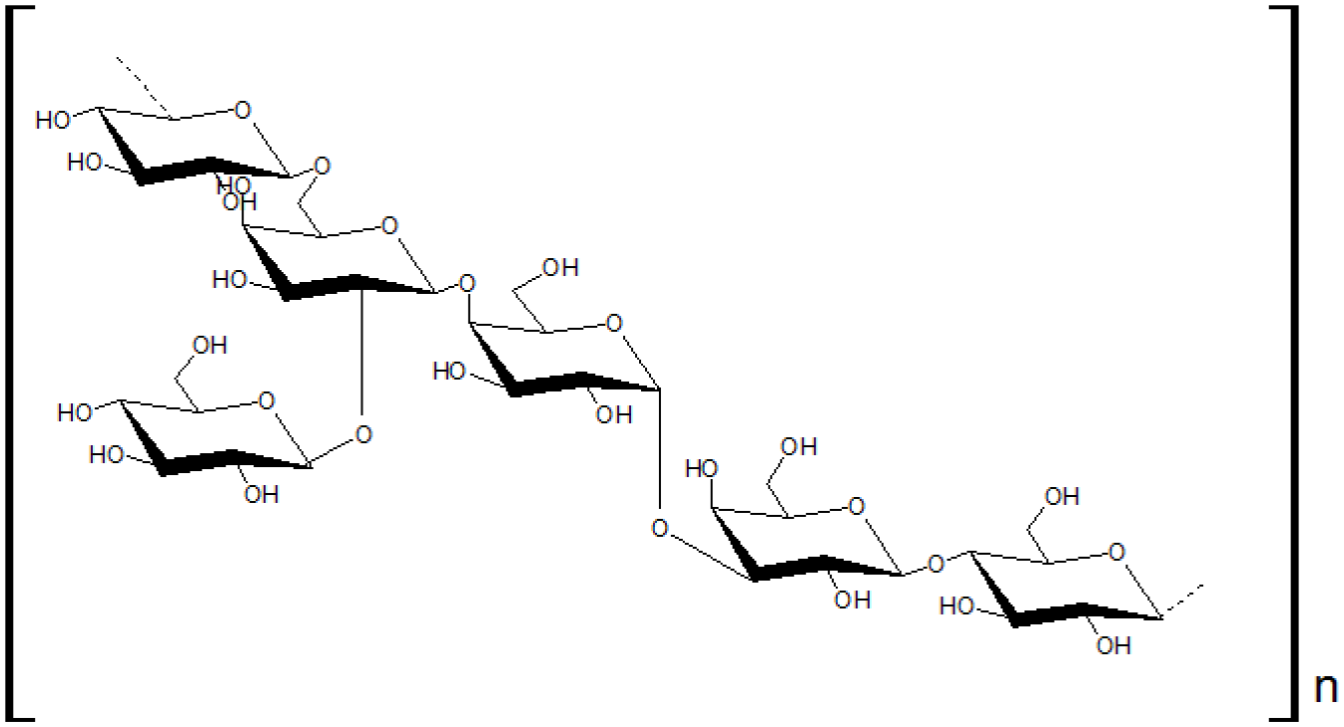
**Kefiran structure**.

The first study about kefiran structure was published by Kooiman (1968), who proposed a structure composed of two units: kefiran (polysaccharide) and kefirose (pentasaccharide). Then, some authors analyzed the polysaccharide structure with current techniques such chromatography and infrared spectroscopy (Wang et al., 2008; Chen et al., 2015) and nuclear magnetic resonance (NMR; Ghasemlou et al., 2012). The kefiran structure, according to them, is shown in **Figure 1**.

## Kefir-Based Products

Nowadays, the interest in developing functional foods is increasing because people want to improve their health and prevent diseases. Keeping in mind that kefir is a beverage with high probiotic activity, among other bioactivities, new companies are emerging around the world. One of the biggest kefir companies known is Lifeway, which started in 1986; their products can be obtained in the United States, Canada, and Great Britain, all of them based in kefir beverages, frozen, and cheese.

Other companies are Evolve Kefir with its principal product, a smoothie; Wallaby Yogurt Company with Low Fat Kefir; and CocoKefir LLC, which provides drinks/beverages based mainly on coconut water cultured with a comprehensive blend of probiotics. **Table 4** summarizes the products provided these companies with some general information about each one.

**Table 4:** Marketed kefir-based products and their information.

Companies	Product	General information
Lifeway • United States • Canada • Great Britain	Low Fat Kefir Non-Fat Kefir Veggie Kefir	All-natural 99% lactose-free Gluten-free 12 probiotic cultures High in protein and calcium
	Kefir Oats	All-natural 99% lactose-free Gluten-free 12 probiotic cultures Oat fiber enriched High in protein and calcium
	Perfect 12 Kefir Traditional Kefir Greek Style Kefir	All-natural 99% lactose-free Gluten-free 12 probiotic cultures No added sugar High in protein and calcium
	Low Fat Kefir (Organic)	USDA Certified Organic Oregon Tilth Certified Organic 99% lactose-free Gluten-free 12 probiotic cultures High in protein and calcium
	Whole Milk Kefir (Organic)	USDA Certified Organic Oregon Tilth Certified Organic 99% lactose-free Gluten-free 12 probiotic cultures No added sugar
	Helios Kefir (Organic)	USDA Certified Organic Oregon Tilth Certified Organic 99% lactose-free Gluten-free Seven probiotic cultures Contains Inulin
	Green Kefir (Organic)	USDA Certified Organic Oregon Tilth Certified Organic 99% lactose-free Gluten-free 12 probiotic cultures Phytoboost = 1 serving of vegetables
	ProBugs (organic)	USDA Certified Organic Oregon Tilth Certified Organic 99% lactose-free Gluten-free 12 probiotic cultures No-spill pouch
	ProBugs Blast (Organic)	USDA Certified Organic Oregon Tilth Certified Organic 99% lactose-free Gluten-free 12 probiotic cultures High in protein and calcium
	Frozen ProBugs (Organic)	All-natural 99% lactose-free Gluten-free 10 probiotic cultures High in protein and calcium
	Frozen Kefir	All-natural 99% lactose-free 10 probiotic cultures 90 calories per serving 1 g of fat
	Frozen Kefir Bars	All-natural 99% lactose-free Gluten-free 10 probiotic cultures 60 calories per serving 0.5 g of fat
	BioKefir	All-natural 20 Billion units of probiotics 12 probiotic cultures 99% lactose-free Gluten-free High in protein and calcium
	Farmer Cheese	99% lactose-free Gluten-free High in protein and calcium
Evolve Kefir • United States	Evolve Kefir	11 probiotic cultures. Natural fruit flavors. Fiber. Protein and calcium
Wallaby Organic • Australia	Lowfat Kefir	12 different strains of Live and Active Kefir cultures.
CocoKefir • United States	CocoKefir App le Cinnamon CocoKefir Citrus CocoKefir CocoYo Body Ecology Coconut Kefir	Dairy, gluten, soy, and fat free Low calorie Contains valuable nutrients such as potassium, manganese, and magnesium. Beneficial probiotic strains

## Conclusion

Kefir, the traditional beverage, is now recognized as a potential source of probiotics and molecules with highly interesting healthy properties. The careful and detailed characterization of kefir composition has helped the scientific community to find new possibilities for its application. Kefiran, the EPS of kefir, has very important physicochemical and rheological properties. Besides, its biological properties suggest its use as antioxidant, antitumor agent, antimicrobial agent, and immunomodulator, among other roles. Research is constantly being conducted to consolidate kefir and kefiran properties for the development of new important products to preserve consumer’s health.

## Acknowledgment

Authors want to thank CNPq and CAPES for the financial support.

## Conflict of Interest Statement

The authors declare that the research was conducted in the absence of any commercial or financial relationships that could be construed as a potential conflict of interest.
